# Association of acupuncture timing and treatment intensity with short-term functional recovery in stroke rehabilitation inpatients: a retrospective cohort and prediction study

**DOI:** 10.3389/fneur.2026.1848746

**Published:** 2026-07-22

**Authors:** Yanling Gao, Jiajun Kang, Ting Rao, Jinhua Lu, Xiaofeng Chen, Qingyan Su, Qing Sun, Qingyue Dai

**Affiliations:** 1 Fujian Key Laboratory of Rehabilitation Technology, Fuzhou, Fujian, China; 2 Fujian University of Traditional Chinese Medicine Subsidiary Rehabilitation Hospital, Fuzhou, Fujian, China; 3 The Second Affiliated Hospital of Fujian University of Traditional Chinese Medicine, Fuzhou, Fujian, China

**Keywords:** acupuncture, Barthel Index, clinical prediction, Fugl-Meyer assessment, machine learning, observational cohort, stroke rehabilitation, treatment intensity

## Abstract

**Background:**

In inpatient stroke rehabilitation settings where acupuncture is part of routine care, several practical questions arise: when treatment should begin, how intensively it should be delivered, and whether data on treatment patterns can help guide individualized discharge planning. We examined treatment-pattern associations in a retrospective acupuncture-treated cohort; lesion-side analyses were treated as secondary exploratory heterogeneity analyses.

**Methods:**

We analyzed 1,048 stroke rehabilitation inpatients who received acupuncture during hospitalization. Median onset-to-first acupuncture was 26 days (IQR 20–35), median total exposure was 20 sessions (IQR 15–26), and median weekly frequency was 5.0 sessions/week (IQR 4.5–5.5). Primary outcomes were admission-to-discharge changes in Barthel Index (BI), Fugl-Meyer Assessment upper extremity (FMA-UE), lower extremity (FMA-LE), and balance; composite recovery was the mean standardized z-score across these four primary change domains. Secondary/exploratory outcomes were Montreal Cognitive Assessment (MoCA) and FMA-ROM/Pain. Home discharge was analyzed as a pragmatic discharge-planning endpoint. Analyses included regression models, subgroup interaction tests, total-session, extended-adjustment, and missingness-oriented sensitivity analyses, inverse-probability weighting, and 3-fold internally validated machine-learning benchmarks.

**Results:**

Patients improved in BI (13.74 +/− 6.57), FMA-UE (10.18 +/− 5.86), FMA-LE (4.90 +/− 3.08), and FMA-balance (2.85 +/− 2.22). Because these instruments have different score ranges, standardized effect sizes and recovery ratios were used to support cross-domain interpretation. Earlier acupuncture initiation was the most consistent favorable exposure signal; for BI gain, the timing coefficient was beta = −1.55 (95% CI −1.96 to −1.14; *p* < 0.001). Mean total sessions were higher among patients discharged home than among non-home patients (21.86 +/− 8.19 vs. 19.60 +/− 7.62; *p* < 0.0001), and total-session models were associated with BI gain (beta = 2.83, 95% CI 2.46–3.20; *p* < 0.001) and home discharge (OR 1.39, 95% CI 1.19–1.62; *p* < 0.001). Left-versus-right interactions were not significant; stroke-type interaction was evident only for timing with FMA-LE gain (*p* = 0.012). In prediction benchmarks, LARE had AUROC 0.804 (95% bootstrap CI 0.778–0.829) for home discharge but was not statistically distinguishable from CatBoost or RandomForest; BI-gain regression was moderate.

**Conclusion:**

Within this acupuncture-treated inpatient cohort, earlier initiation, weekly frequency, and total exposure were associated with short-term ADL and motor recovery. These findings are consistent with timing- and dose–response hypotheses from the broader rehabilitation and acupuncture literature, but prospective studies with non-acupuncture comparators and acute neurologic severity measures are needed before causal efficacy claims can be made.

## 1 Introduction

Stroke remains one of the leading causes of death and long-term disability worldwide (1). Contemporary rehabilitation guidelines emphasize coordinated, multidisciplinary care delivered across the continuum from acute hospitalization to inpatient and community rehabilitation (2, 3). In parallel, the stroke-rehabilitation literature has increasingly focused on timing and dose, because recovery is influenced not only by what therapy is provided, but also by when it begins and how intensively it is delivered (4–7). Stroke rehabilitation is inherently multimodal. In many real-world inpatient systems, acupuncture is not delivered as an isolated intervention but as one component of a broader program that also includes physical therapy, occupational therapy, speech therapy, medications, and routine nursing care. In such settings, a simple “acupuncture versus no acupuncture” question may be less informative than a more practical question: within an acupuncture-integrated rehabilitation pathway, which treatment patterns are associated with better short-term recovery?

That distinction matters for this dataset. The present cohort is a retrospective hospital-based series of stroke rehabilitation inpatients who all received acupuncture-related treatment. Accordingly, the most defensible clinical framing is not comparative efficacy, but optimization of treatment timing and intensity inside an acupuncture-treated population. Existing acupuncture-after-stroke literature remains heterogeneous: earlier reviews and meta-analyses have reported mixed functional findings and repeatedly emphasized limited study quality, protocol heterogeneity, and uncertainty about causal efficacy (8–12). A recent dose-effect systematic review specifically examined acupuncture dose for post-stroke limb dysfunction, but the broader literature still summarizes highly variable intervention packages and provides limited evidence on clinically actionable start-time and weekly-frequency decisions in routine inpatient care (13).

A second limitation of many retrospective acupuncture papers is that they often stop at crude pre-post comparisons. Conversely, prediction studies in stroke rehabilitation increasingly use machine-learning algorithms, but they are often framed as general prognostic exercises rather than as analyses tied to a specific modifiable clinical question such as acupuncture timing, weekly treatment density, lesion-side heterogeneity, or discharge-planning use (14–17). Recent reviews and derivation studies suggest that prediction modeling in stroke rehabilitation is growing rapidly, but models remain heterogeneous in inputs, outcomes, validation strategies, and clinical targets (14–17). We aimed to connect these components. Specifically, we examined whether earlier initiation and higher weekly acupuncture frequency were associated with larger gains in activities of daily living (ADL) and motor recovery, whether these associations differed by lesion laterality and stroke type, and whether the heterogeneity pattern could be translated into a clinically useful prediction framework.

We therefore designed the manuscript around three linked aims: (1) characterize whole-cohort inpatient recovery and the main treatment-pattern associations; (2) explore secondary heterogeneity by lesion side, stroke type, and lesion anatomy without treating nonsignificant left–right contrasts as definitive effect modification; and (3) build internally validated prediction benchmarks for home discharge and BI improvement that include lesion side as a clinical feature and explicitly test, rather than assume, the incremental value of laterality-aware modeling.

## 2 Materials and methods

### 2.1 Study design and data source

This study used a retrospective single-center observational cohort derived from the Fujian University of Traditional Chinese Medicine Subsidiary Rehabilitation Hospital. The source file contained 1,048 stroke rehabilitation inpatients admitted between January 2023 and September 2024 who received acupuncture during the inpatient stay. The working dataset included demographics, dates of admission and discharge, stroke type, lesion location, hemiplegic side, functional assessments, acupuncture treatment descriptors, total acupuncture session counts, rehabilitation intensity, comorbidities, medications, acute-phase intervention history, and discharge destination. NIHSS at stroke onset or at acupuncture initiation was not available in the exported source data; baseline BI and FMA scores at rehabilitation admission were therefore used as functional severity measures, not as substitutes for NIHSS.

### 2.2 Participants and variable engineering

The analytic cohort included patients with ischemic stroke or intracerebral hemorrhage who underwent inpatient rehabilitation with acupuncture-related treatment. To support clinically interpretable subgroup analysis, lesion side was collapsed into left, right, bilateral, and mixed categories, and lesion anatomy was collapsed into subcortical, cortical, cortico-subcortical, brainstem/cerebellum, and mixed/posterior-fossa groups. Treatment-related variables focused on onset-to-first acupuncture timing, weekly acupuncture frequency, total acupuncture sessions, electroacupuncture exposure, and broader treatment-component composition. Weekly frequency was retained as the main intensity variable because it normalizes treatment density to different lengths of stay, whereas total sessions was examined in sensitivity analyses because it also reflects inpatient exposure duration. Rehabilitation intensity was derived from physical therapy (PT), occupational therapy (OT), and speech therapy (ST) session information in the source file; total PT/OT/ST sessions and sessions per week were calculated from recorded session counts and length of stay. Treatment-component count was defined as the number of acupuncture-related modalities recorded for a patient, including body/manual acupuncture, scalp acupuncture, electroacupuncture, Tuina, gua sha, auricular acupuncture, embedded-needle therapy, and warm-needle moxibustion; a higher count therefore indicates a more diverse acupuncture-related treatment package rather than a higher dose of any single modality. Comorbidity burden was defined as the count of recorded comorbid conditions in the source file. Discharge destination was dichotomized as home versus non-home for pragmatic discharge-planning analyses.

### 2.3 Outcome definitions

Primary outcomes were admission-to-discharge change scores for BI, FMA-UE, FMA-LE, and FMA-balance. These four primary domains were fully observed in all 1,048 patients and therefore carried the main inferential weight. They were selected because the Barthel Index captures dependence in activities of daily living (18), and the Fugl-Meyer system provides standardized quantitative assessment of post-stroke sensorimotor impairment, including upper-extremity, lower-extremity, balance, and joint-function domains (19, 20). Secondary outcomes were MoCA and FMA-ROM/Pain. The Montreal Cognitive Assessment is a brief cognitive screening tool that has been widely adopted in stroke populations (21–23). Because MoCA and ROM/Pain were incompletely captured, they were prespecified and interpreted as supportive/exploratory outcomes rather than co-primary endpoints. Discharge destination was analyzed as a pragmatic discharge-planning endpoint because it is clinically relevant to rehabilitation pathways, but it was not treated as a direct recovery measure; it can be influenced by premorbid support, transfer policy, nursing-home availability, and length of stay. Composite recovery was calculated as the average of the standardized z-scores for BI, FMA-UE, FMA-LE, and FMA-balance change, so that a positive value indicated above-average recovery across the four fully observed primary domains. Because admission-to-discharge change scores can be affected by length of stay and exposure opportunity, length-of-stay and rehabilitation-intensity variables were considered in sensitivity analyses and are emphasized in the limitations.

### 2.4 Statistical analysis

We first summarized baseline characteristics and discharge disposition and then assessed admission-to-discharge change using paired analyses. Multiple linear regression was used for continuous change outcomes, and logistic regression was used for home discharge. The selected whole-cohort coefficients reported in Table 1 come from the core treatment-pattern model, which included timing_z, acu_freq_z, baseline_motor_z, hemorrhage, age_z, comorb_z, and electroacupuncture exposure, but did not include lesion side as an additional covariate. Lesion side was instead handled as a prespecified heterogeneity and prediction variable: it was evaluated through stratified left/right models, formal interaction models, extended sensitivity models that included a right-lesion indicator, and prediction models that included lesion side as a model feature. For visualization, timing, weekly frequency, and total sessions were also grouped into quartiles. Prespecified subgroup analyses evaluated lesion laterality and stroke type, with anatomy-level descriptive contrasts shown separately. Formal interaction models tested timing-by-group, frequency-by-group, and electroacupuncture-by-group terms for lesion side and stroke type. Exploratory timing-by-frequency interaction models tested whether weekly treatment density modified the timing association. Total-session sensitivity analyses used two specifications. The total-session model included timing_z, acu_total_z, baseline_motor_z, hemorrhage, age_z, comorb_z, and electroacupuncture exposure. The joint frequency plus total-session model included timing_z, acu_freq_z, acu_total_z, baseline_motor_z, hemorrhage, age_z, comorb_z, and electroacupuncture exposure. Extended sensitivity models retained timing_z and acu_freq_z and added the broader covariate set available in the processed dataset: baseline_motor_z, age_z, comorb_z, hemorrhage, electroacupuncture exposure, sex, right-lesion indicator, length-of-stay z score, rehabilitation-sessions/week z score, acute intervention indicator, acupuncture component count, and lesion-anatomy indicators. For secondary outcomes, observation models first estimated the probability that paired MoCA or ROM/Pain data were observed using timing_z, acu_freq_z, baseline_motor_z, electroacupuncture exposure, age_z, hemorrhage, sex, right-lesion indicator, rehabilitation-sessions/week z score, and length-of-stay z score; inverse-probability weights were then used in observation-IPW sensitivity analyses to reduce bias from incomplete observations under plausible missing-at-random structures (24). For the right-lesion electroacupuncture signal, the unadjusted model included electroacupuncture alone; the base-adjusted model added timing, weekly frequency, baseline motor severity, age, comorbidity burden, and hemorrhage status; the rehabilitation-intensity-adjusted model additionally included rehabilitation sessions per week and length of stay; the baseline-domain-adjusted model additionally included admission BI and FMA domain scores; anatomy/component models additionally included lesion-anatomy and acupuncture-component variables; and IPTW models weighted by the inverse probability of receiving electroacupuncture. All reported *p* values were two-sided.

**Table 1:** Selected whole-cohort multivariable coefficients for continuous outcomes, composite recovery, and home discharge.

Outcome	Predictor	Coefficient	CI low	CI high	*p* value
BI gain	timing z	−1.546	−1.956	−1.137	<0.0001
BI gain	frequency z	1.170	0.777	1.563	<0.0001
BI gain	baseline motor z	−0.970	−1.413	−0.527	<0.0001
BI gain	hemorrhage	−1.197	−2.144	−0.249	0.013
BI gain	age z	−0.438	−0.821	−0.056	0.025
BI gain	comorbidity z	−0.375	−0.751	0.001	0.050
BI gain	electro	−0.826	−1.684	0.033	0.059
FMA-UE gain	timing z	−0.565	−0.870	−0.260	0.0003
FMA-UE gain	frequency z	0.582	0.222	0.941	0.002
FMA-UE gain	baseline motor z	−0.959	−1.382	−0.536	<0.0001
FMA-UE gain	hemorrhage	−0.947	−1.865	−0.029	0.043
FMA-UE gain	age z	−0.100	−0.463	0.263	0.590
FMA-UE gain	comorbidity z	−0.194	−0.549	0.161	0.285
FMA-UE gain	electro	−0.159	−0.949	0.632	0.694
FMA-LE gain	timing z	−0.410	−0.580	−0.239	<0.0001
FMA-LE gain	frequency z	0.317	0.128	0.506	0.001
FMA-LE gain	baseline motor z	−0.608	−0.816	−0.399	<0.0001
FMA-LE gain	hemorrhage	−0.602	−1.084	−0.120	0.014
FMA-LE gain	age z	−0.106	−0.293	0.081	0.268
FMA-LE gain	comorbidity z	−0.089	−0.274	0.096	0.346
FMA-LE gain	electro	−0.264	−0.683	0.156	0.218
FMA-balance gain	timing z	−0.244	−0.380	−0.108	0.0004
FMA-balance gain	frequency z	0.051	−0.085	0.187	0.464
FMA-balance gain	baseline motor z	−0.426	−0.579	−0.272	<0.0001
FMA-balance gain	hemorrhage	−0.442	−0.790	−0.094	0.013
FMA-balance gain	age z	−0.065	−0.199	0.070	0.347
FMA-balance gain	comorbidity z	−0.131	−0.267	0.006	0.061
FMA-balance gain	electro	−0.205	−0.505	0.095	0.181
composite recovery	timing z	−0.102	−0.142	−0.062	<0.0001
composite recovery	frequency z	0.101	0.060	0.142	<0.0001
composite recovery	baseline motor z	0.445	0.393	0.497	<0.0001
composite recovery	hemorrhage	−0.126	−0.213	−0.039	0.005
composite recovery	age z	−0.035	−0.075	0.005	0.089
composite recovery	comorbidity z	−0.041	−0.079	−0.003	0.035
composite recovery	electro	−0.090	−0.178	−0.002	0.046
home discharge OR	baseline motor z	4.357	3.551	5.346	<0.0001
home discharge OR	frequency z	1.283	1.106	1.490	0.001
home discharge OR	timing z	0.957	0.825	1.110	0.560
home discharge OR	hemorrhage	0.736	0.511	1.059	0.099
home discharge OR	age z	0.947	0.816	1.099	0.471
home discharge OR	comorbidity z	0.876	0.758	1.013	0.074
home discharge OR	electro	0.900	0.650	1.248	0.528

Continuous outcomes are reported as beta estimates with 95% CI; home discharge is reported as odds ratios (ORs) with 95% CI. timing z and frequency z denote standardized timing and weekly-frequency terms from the core whole-cohort model. Lesion side is not included in the table core model; it is evaluated in stratified, interaction, extended-sensitivity, and predictive models.

### 2.5 Predictive modeling

We benchmarked multiple supervised learning approaches for two clinically relevant tasks: prediction of home discharge and prediction of BI improvement. The tested models were ElasticNet/ElasticNet-logit, RandomForest, multilayer perceptron (MLP), CatBoost gradient-boosted trees, and an exploratory laterality-aware residual ensemble (LARE). ElasticNet was used as a regularized linear baseline (25), RandomForest as a bagged decision-tree ensemble (26), MLP as a feed-forward neural-network model (27), and CatBoost as a gradient-boosted tree approach related to the XGBoost boosting family (28, 29). All predictive models used the same prespecified feature set: age, sex, stroke type, lesion side, anatomy group, lesion location, hemiplegic side, length of stay, onset-to-first acupuncture timing, acupuncture sessions per week, rehabilitation sessions per week, admission BI, admission FMA-UE, admission FMA-LE, admission FMA-balance, admission MoCA, admission FMA-ROM/Pain, comorbidity count, hypertension, diabetes mellitus, atrial fibrillation, hyperlipidemia, cerebral atherosclerosis, coronary artery disease, any acute intervention, intervention category, electroacupuncture, Tuina, auricular acupuncture, warm-needle moxibustion, gua sha, embedded-needle therapy, acupuncture component count, and speech therapy use. LARE was an internally specified benchmark used to test whether side-specific expert blending added incremental predictive value, not a previously named external algorithm or a prespecified primary methodologic contribution. In each training fold, a global CatBoost model was fit using the full feature set, including lesion side; separate left- and right-lesion expert CatBoost models were then trained within the corresponding lesion-side subsets when at least 30 training observations were available. For left- or right-lesion test patients, the final LARE prediction was calculated as 0.65 x global prediction + 0.35 x side-specific expert prediction; for bilateral/mixed lesions or sparse expert strata, the global prediction was retained. The same blending rule was used for home-discharge classification and BI-gain regression. Model performance was estimated by 3-fold internal validation: stratified K-fold cross-validation for home-discharge classification and K-fold cross-validation for BI-gain regression. Performance was summarized using AUROC, AUPRC, and Brier score for home-discharge classification, and R2, RMSE, and MAE for BI-gain regression. Nonparametric bootstrap of out-of-fold predictions with 2,000 resamples was used to estimate 95% confidence intervals and pairwise *p* values for model-metric differences; classification comparisons used LARE as the reference because it had the highest point estimates, whereas regression comparisons used RandomForest as the reference because it had the strongest point-estimate regression performance. We emphasized discrimination, overall performance, and calibration in line with recommended prediction-model evaluation frameworks (30–33), and reported the modeling work in the spirit of contemporary guidance for regression- and machine-learning-based clinical prediction models (34, 35). Calibration and clinical-utility metrics, including calibration intercept, slope, expected calibration error, and decision-curve net benefit, are reported in Supplementary Table S7 and Supplementary Figure S3.

## 3 Results

### 3.1 Cohort characteristics and rehabilitation context

**Figure 1:**
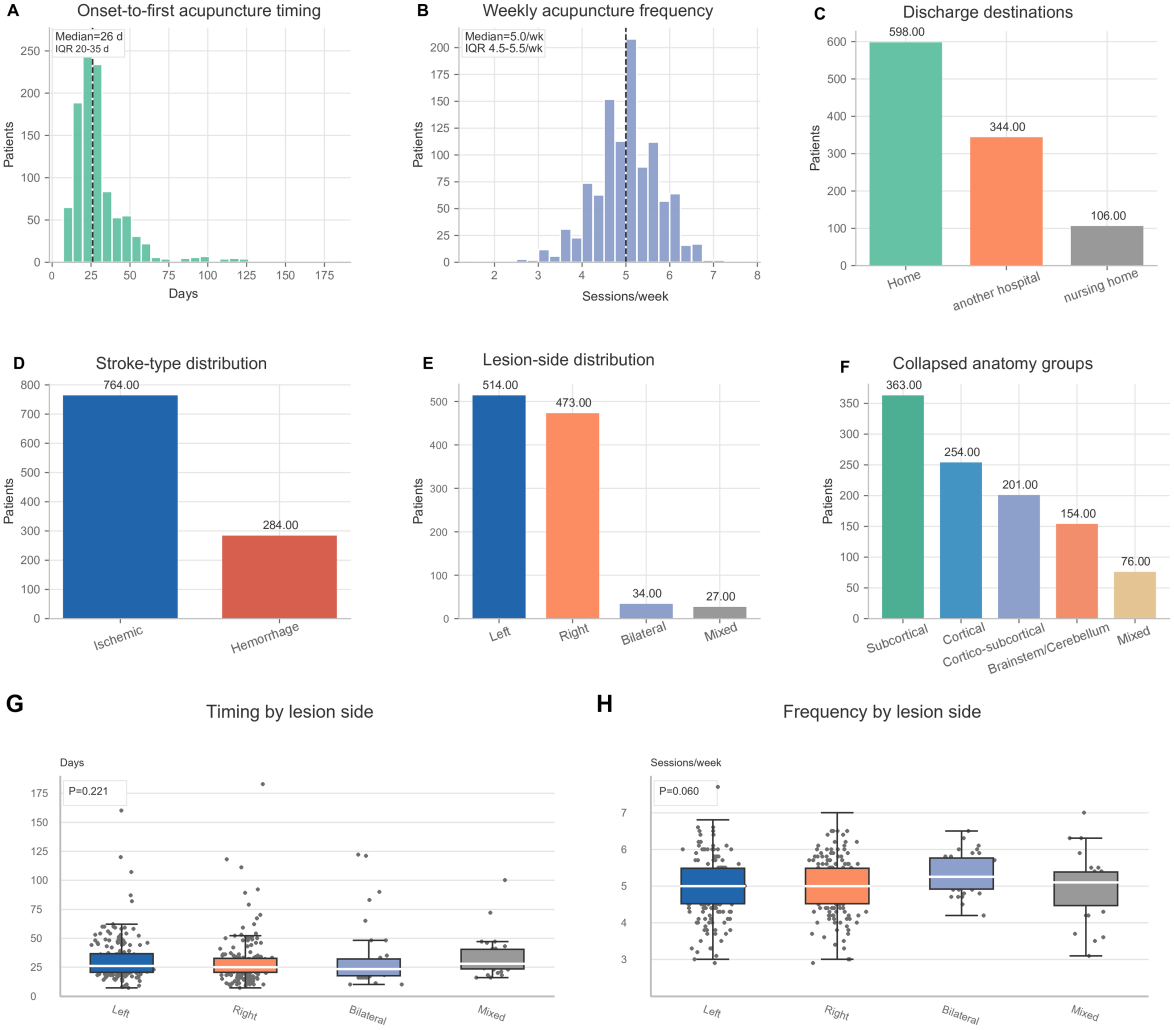
Cohort overview and treatment exposure distributions. **(A)** Histogram of onset-to-first acupuncture timing, showing a median interval of 26 days (IQR 20–35). **(B)** Histogram of weekly acupuncture frequency, centered at a median of 5.0 sessions/week (IQR 4.5–5.5). **(C)** Distribution of discharge destinations, with 598 patients discharged home, 344 transferred to another hospital, and 106 transferred to a nursing home. **(D–F)** Cohort composition by stroke type (764 ischemic, 284 hemorrhagic), lesion side (514 left, 473 right, 34 bilateral, and 27 mixed), and collapsed anatomy group (363 subcortical, 254 cortical, 201 cortico-subcortical, 154 brainstem/cerebellum, and 76 mixed/posterior-fossa). **(G)** Onset-to-first acupuncture timing was broadly similar across lesion-side groups (*p* = 0.221). **(H)** Weekly acupuncture frequency was broadly similar across lesion-side groups (*p* = 0.060). Together, these panels define the real-world acupuncture-treated rehabilitation context and directly display treatment exposure by lesion side.

**Figure 2:**
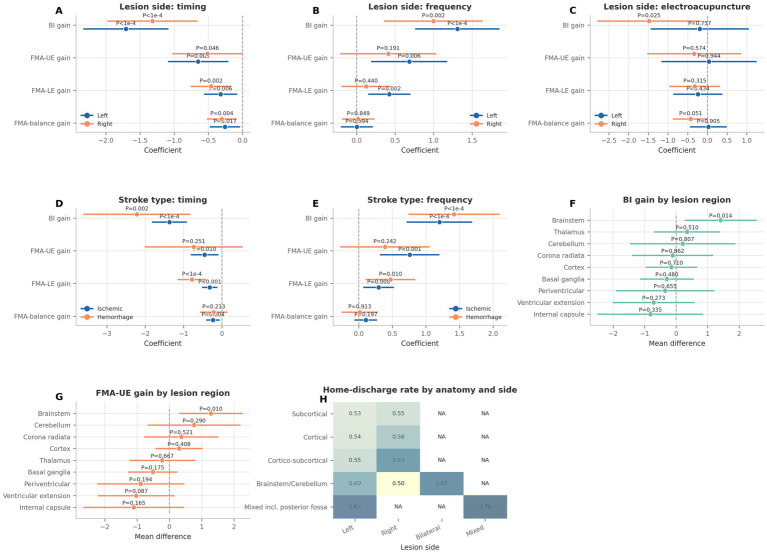
Focused heterogeneity analyses by lesion side, stroke type, and lesion anatomy. **(A-C)** Lesion-side-stratified coefficients for timing, weekly frequency, and electroacupuncture across BI, FMA-UE, FMA-LE, and FMA-balance gain. **(D,E)** Stroke-type-stratified timing and weekly-frequency coefficients across the same outcomes. **(F,G)** Region-present versus region-absent mean differences for BI and FMA-UE gain, respectively. **(H)** Home-discharge rate by collapsed anatomy group and lesion side, displayed with a muted shared color scale over the narrow observed range. The figure is descriptive; formal coefficient-difference interaction tests are reported in Supplementary Table S9. Left-versus-right interaction terms were not statistically significant, whereas the stroke-type interaction was significant only for the timing association with FMA-LE gain. Anatomy-by-side discharge-rate contrasts are shown to describe small-stratum patterns, not to claim statistically confirmed anatomy-specific effects.

The cohort comprised 1,048 acupuncture-treated stroke rehabilitation inpatients, with a mean age of 65.73 +/− 10.72 years; 608 patients (58.0%) were male (Table 2). Ischemic stroke accounted for 764 cases (72.9%) and intracerebral hemorrhage for 284 cases (27.1%). Lesion laterality was nearly balanced between left-sided (514, 49.0%) and right-sided (473, 45.1%) disease, with smaller bilateral and mixed groups. Home discharge occurred in 598 patients (57.1%), whereas 344 were transferred to another hospital and 106 to a nursing home (Table 2; Figures 1C–E). Exposure distributions were consistent with a routine inpatient rehabilitation pathway. The median onset-to-first acupuncture interval was 26 days (IQR 20–35), the median total number of acupuncture sessions was 20 (IQR 15–26), and the median weekly acupuncture frequency was 5.0 sessions/week (IQR 4.5–5.5; Figures 1A,B; Table 2). Mean total sessions were higher in the home-discharge group than in the non-home group (21.86 +/− 8.19 vs. 19.60 +/− 7.62; *p* < 0.0001). Total sessions correlated strongly with length of stay (Spearman rho = 0.93) and moderately with weekly frequency (rho = 0.50), so total sessions was treated as a sensitivity exposure rather than as a replacement for the primary weekly-frequency term. Timing and weekly-frequency distributions were broadly similar across lesion-side groups (timing *p* = 0.221; frequency *p* = 0.060), suggesting that gross exposure imbalance by lesion side was limited (Figures 1G,H). Baseline differences by discharge destination were clinically meaningful: patients discharged home had better admission BI and FMA scores, slightly higher weekly acupuncture frequency, more total acupuncture sessions, and a slightly longer length of stay, whereas age and onset-to-first acupuncture timing were similar between home and non-home groups (Table 2). Quality-control plots showed that change scores across motor and ADL domains were moderately correlated but not redundant, while timing and frequency distributions by stroke type appeared stable enough to support subgroup comparisons (Supplementary Figure S1). Detailed lesion-region prevalence and present-versus-absent contrasts are shown in Figures 2F–H and Supplementary Figure S2.

**Table 2:** Baseline characteristics, acupuncture exposure, and discharge-stratified cohort profile.

Variable	Overall	Home discharge	Non-home discharge	*p* value
Age	65.73 ± 10.72	65.11 ± 10.34	66.55 ± 11.17	0.093
Length of Stay (days)	29.06 ± 9.73	30.05 ± 9.84	27.74 ± 9.45	<0.0001
Onset-to-Rehab Admission Interval (days)	29.25 ± 20.65	28.99 ± 20.79	29.60 ± 20.47	0.151
Onset-to-First Acupuncture (days)	31.09 ± 20.75	30.75 ± 20.89	31.54 ± 20.58	0.140
Total Acupuncture Sessions	20.89 +/− 8.03	21.86 +/− 8.19	19.60 +/− 7.62	<0.0001
Acupuncture Sessions/Week	4.99 ± 0.74	5.05 ± 0.73	4.91 ± 0.73	0.003
Rehab Sessions/Week	12.15 ± 2.08	12.20 ± 2.08	12.08 ± 2.07	0.299
BI at Admission	43.85 ± 18.44	52.42 ± 15.38	32.46 ± 15.82	<0.0001
FMA Upper Extremity at Admission	28.77 ± 14.84	34.46 ± 13.42	21.20 ± 13.17	<0.0001
FMA Lower Extremity at Admission	15.08 ± 7.32	17.71 ± 6.74	11.59 ± 6.56	<0.0001
FMA Balance at Admission	10.73 ± 5.22	12.67 ± 4.76	8.14 ± 4.66	<0.0001
Sex: Male	608 (58.0%)	342 (57.2%)	266 (59.1%)	
Sex: Female	440 (42.0%)	256 (42.8%)	184 (40.9%)	
Stroke Type: Ischemic stroke	764 (72.9%)	479 (80.1%)	285 (63.3%)	
Stroke Type: Intracerebral hemorrhage	284 (27.1%)	119 (19.9%)	165 (36.7%)	
Lesion Side: Left	514 (49.0%)	287 (48.0%)	227 (50.4%)	
Lesion Side: Right	473 (45.1%)	270 (45.2%)	203 (45.1%)	
Lesion Side: Bilateral	34 (3.2%)	22 (3.7%)	12 (2.7%)	
Lesion Side: Mixed	27 (2.6%)	19 (3.2%)	8 (1.8%)	
Anatomy Group: Brainstem/Cerebellum	154 (14.7%)	88 (14.7%)	66 (14.7%)	
Anatomy Group: Cortical	254 (24.2%)	140 (23.4%)	114 (25.3%)	
Anatomy Group: Cortico-subcortical	201 (19.2%)	121 (20.2%)	80 (17.8%)	
Anatomy Group: Mixed incl. Posterior fossa	76 (7.3%)	52 (8.7%)	24 (5.3%)	
Anatomy Group: Subcortical	363 (34.6%)	197 (32.9%)	166 (36.9%)	
Hemiplegic Side: Left	483 (46.1%)	273 (45.7%)	210 (46.7%)	
Hemiplegic Side: Right	565 (53.9%)	325 (54.3%)	240 (53.3%)	
Discharge Destination: Home	598 (57.1%)	598 (100.0%)	0 (0.0%)	
Discharge Destination: Transferred to another hospital	344 (32.8%)	0 (0.0%)	344 (76.4%)	
Discharge Destination: Transferred to nursing home	106 (10.1%)	0 (0.0%)	106 (23.6%)	

Continuous data are presented as mean +/− SD; categorical values are n (%). Non-home discharge includes transfer to another hospital or nursing home. *p* values are shown where provided in the finalized table export. Total acupuncture sessions is reported as an exposure summary.

### 3.2 Admission-to-discharge functional recovery

**Figure 3:**
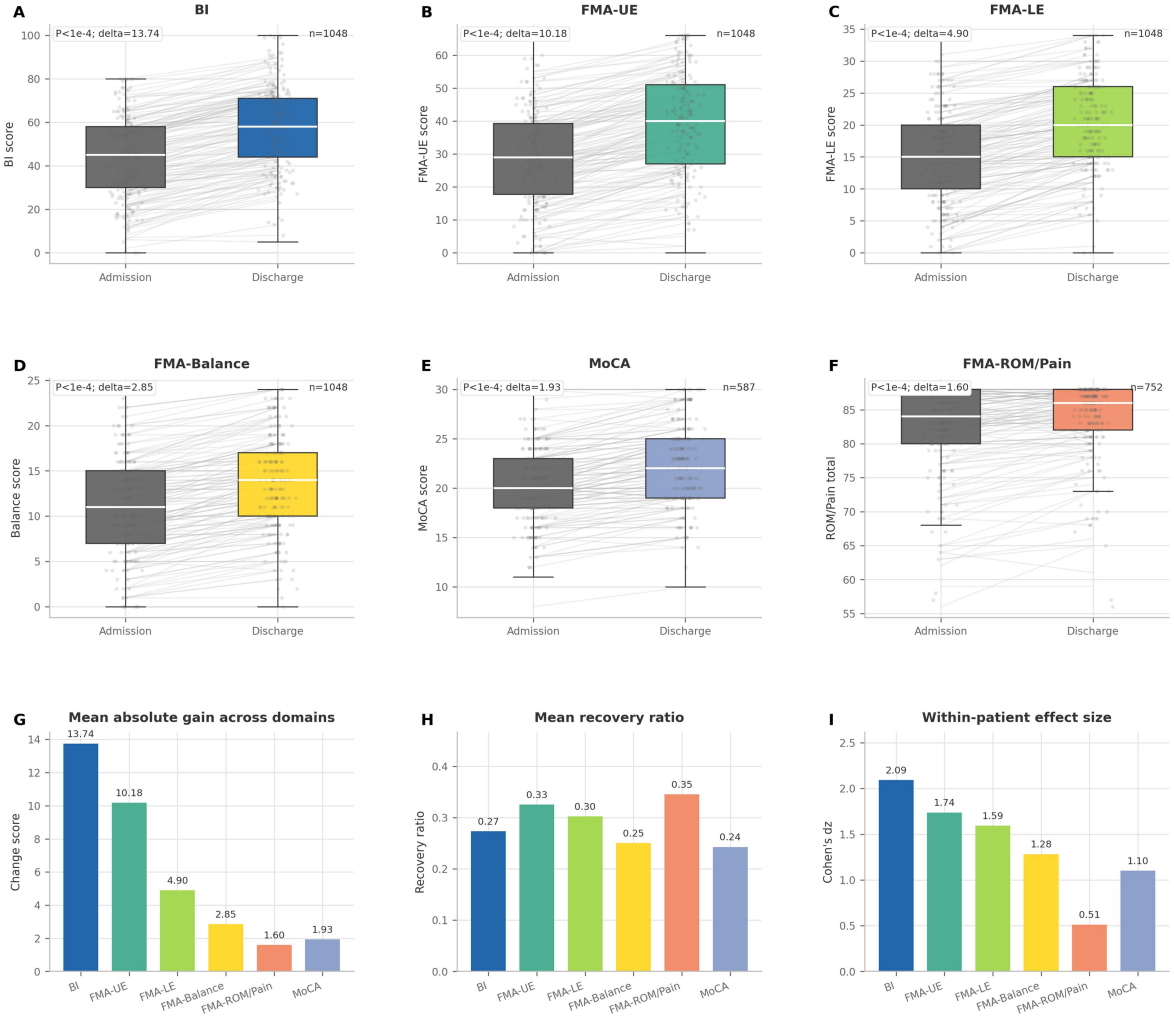
Admission-to-discharge improvement across core functional domains. **(A–F)** Paired admission-versus-discharge plots for BI, FMA-UE, FMA-LE, FMA-balance, MoCA, and FMA-ROM/Pain, respectively. All six domains improved significantly during inpatient rehabilitation (all paired *p* < 1 × 10^−4^), with mean changes of +13.74 for BI, +10.18 for FMA-UE, +4.90 for FMA-LE, +2.85 for FMA-balance, +1.93 for MoCA (*n* = 587), and +1.60 for FMA-ROM/Pain (*n* = 752). **(G)** Mean absolute gains across domains. **(H)** Mean recovery ratios across domains. **(I)** Within-patient effect sizes, showing the largest short-term signal in BI and motor recovery. The figure demonstrates that ADL and motor outcomes improved most strongly, whereas cognitive and ROM/Pain gains were smaller and based on incompletely observed subsets.

All major outcomes improved during the inpatient stay (Table 3; Figure 3). On their native scales, mean changes were 13.74 +/− 6.57 for BI, 10.18 +/− 5.86 for FMA-UE, 4.90 +/− 3.08 for FMA-LE, and 2.85 +/− 2.22 for FMA-balance; these absolute values should not be compared directly across instruments because the score ranges differ. Scale-aware interpretation was therefore based on paired effect sizes and recovery ratios. Effect sizes were largest for BI (Cohen’s dz. = 2.09) and the main motor domains (FMA-UE dz. = 1.74; FMA-LE dz. = 1.59; FMA-balance dz. = 1.28), indicating marked within-stay improvement in ADL and motor function (Table 3; Figures 3A–D,G). Secondary domains also improved, but the signals were comparatively weaker and based on fewer observed patients. Mean MoCA increased by 1.93 +/− 1.75 in the 587 patients with paired assessments, and ROM/Pain increased by 1.60 +/− 3.12 in the 752 patients with paired observations (Table 3; Figures 3E,F). Baseline-severity and recovery-reserve analyses are provided in Supplementary Figure S7 to contextualize raw gain patterns. This pattern supported our outcome hierarchy: BI and FMA changes constituted the primary analytic backbone, whereas MoCA and ROM/Pain were retained as supportive domains only.

**Table 3:** Admission-to-discharge outcomes, paired-comparison significance, and effect sizes.

Outcome	N	Admission mean ± SD	Discharge mean ± SD	Change mean ± SD	Paired t-test *p*	Cohen’s dz	Mean recovery ratio
BI	1,048	43.85 ± 18.44	57.59 ± 18.85	13.74 ± 6.57	<0.0001	2.091	0.273
FMA-UE	1,048	28.77 ± 14.84	38.95 ± 15.29	10.18 ± 5.86	<0.0001	1.738	0.325
FMA-LE	1,048	15.08 ± 7.32	19.99 ± 7.44	4.90 ± 3.08	<0.0001	1.594	0.302
FMA-Balance	1,048	10.73 ± 5.22	13.58 ± 5.38	2.85 ± 2.22	<0.0001	1.283	0.250
FMA-ROM/Pain	752	82.26 ± 6.20	83.86 ± 5.36	1.60 ± 3.12	<0.0001	0.512	0.345
MoCA	587	20.31 ± 4.00	22.23 ± 4.27	1.93 ± 1.75	<0.0001	1.100	0.243

Recovery ratio was calculated at the patient level as (discharge score - admission score) / (scale maximum - admission score), then averaged by outcome. Scale maxima were BI 100, FMA-UE 66, FMA-LE 34, FMA-balance 24, FMA-ROM/Pain 88, and MoCA 30. BI and all FMA domains were fully observed; MoCA and ROM/Pain were observed in subsets only.

### 3.3 Whole-cohort treatment-pattern associations

**Figure 4:**
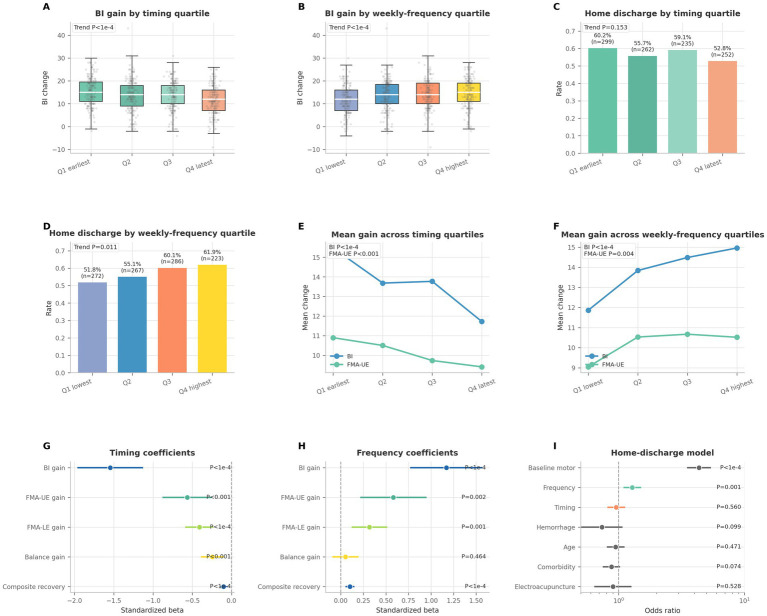
Whole-cohort associations of acupuncture timing and weekly intensity with recovery and discharge. **(A,B)** BI gain across onset-to-first acupuncture timing quartiles and weekly-frequency quartiles, respectively. **(C,D)** Home-discharge rate across timing and weekly-frequency quartiles. **(E,F)** Mean BI and FMA-UE gains across timing and weekly-frequency quartiles. These descriptive panels are presented before adjusted modeling results. **(G)** Standardized coefficients for onset-to-first acupuncture timing across BI gain, FMA-UE gain, FMA-LE gain, balance gain, and composite recovery. Negative coefficients indicate that earlier initiation was associated with larger improvement. **(H)** Standardized coefficients for weekly acupuncture frequency across the same continuous outcomes, showing the strongest favorable frequency signals for BI, FMA-UE, FMA-LE, and composite recovery, but not for balance. **(I)** Fully adjusted home-discharge model, in which baseline motor status was the strongest predictor and higher weekly acupuncture frequency remained independently associated with home discharge, whereas timing was not significant after multivariable adjustment. Overall, the figure shows descriptive exposure gradients first, followed by adjusted associations.

Quartile-based descriptive visualizations were reviewed before adjusted models (Figures 4A–F). BI gain decreased across later timing quartiles (trend *p* < 0.001) and increased across higher weekly-frequency quartiles (trend *p* < 0.001), while home discharge improved across weekly-frequency quartiles (trend *p* = 0.011) but not across timing quartiles (trend *p* = 0.153). FMA-UE and FMA-LE quartile trends followed the same general direction for timing and weekly frequency, although the magnitude was smaller than for BI. Exploratory timing-by-frequency interaction models did not show statistically significant interaction terms for BI gain, FMA-UE gain, FMA-LE gain, FMA-balance gain, composite recovery, or home discharge (Supplementary Table S10; all *p* > = 0.058), so combined timing/frequency patterns were treated as descriptive rather than as evidence of a synergistic exposure effect. In the adjusted multiple linear-regression models, earlier acupuncture initiation was the most stable favorable association across continuous outcomes (Table 1; Figures 4G–I). For BI improvement, the standardized timing coefficient was beta = −1.546 (95% CI −1.956 to −1.137; *p* < 0.001). Timing remained significant for FMA-UE change (beta = −0.565; *p* < 0.001), FMA-LE change (beta = −0.410; *p* < 0.001), balance change (beta = −0.244; *p* < 0.001), and composite recovery (beta = −0.102; *p* < 0.001; Table 1; Figure 4G). Higher weekly acupuncture frequency was associated with BI, FMA-UE, FMA-LE, and composite recovery, but not with balance: BI beta = 1.170 (*p* < 0.001), FMA-UE beta = 0.582 (*p* = 0.002), FMA-LE beta = 0.317 (*p* = 0.001), composite recovery beta = 0.101 (*p* < 0.001), and balance *p* = 0.464 (Table 1; Figure 4H). The practical endpoint of home discharge showed a related but not identical pattern. Baseline motor status was the strongest predictor (OR 4.36, 95% CI 3.55–5.35; *p* < 0.001), but weekly acupuncture frequency remained independently associated with home discharge (OR 1.28, 95% CI 1.11–1.49; *p* = 0.001), whereas timing was not significant in the fully adjusted home-discharge model (OR 0.96; *p* = 0.560; Table 1; Figure 4I). Total-session sensitivity analyses examined whether overall treatment exposure was more informative than weekly frequency alone. In the total-session model, total sessions were associated with BI gain (beta = 2.83, 95% CI 2.46 to 3.20; *p* < 0.001), FMA-UE gain, FMA-LE gain, balance gain, composite recovery, and home discharge (OR 1.39, 95% CI 1.19 to 1.62; *p* < 0.001). In the joint weekly-frequency plus total-session model, total sessions remained associated with BI gain and home discharge, whereas the weekly-frequency estimates attenuated for BI gain (*p* = 0.364) and home discharge (*p* = 0.140), consistent with shared information between treatment density and exposure duration (Supplementary Table S8). Extended sensitivity analyses supported the robustness of the timing signal. After adding broader adjustment terms, the timing association remained significant for BI, FMA-UE, FMA-LE, balance, composite recovery, and retained the same direction for home discharge, whereas the frequency signal attenuated for FMA-UE and FMA-LE but remained significant for BI, composite recovery, and home discharge (Supplementary Table S4).

### 3.4 Heterogeneity by lesion side, stroke type, and anatomy

Focused heterogeneity analyses are summarized in Figure 2 and Supplementary Table S9. In lesion-side-stratified models, earlier initiation remained favorable in both left- and right-sided lesions for BI, FMA-UE, FMA-LE, and balance outcomes, and higher weekly frequency appeared numerically more stable in left-sided lesions (Figures 2A,B). However, formal interaction tests did not show statistically significant left-versus-right differences in timing, frequency, or electroacupuncture coefficients for BI, FMA-UE, FMA-LE, or balance outcomes (all interaction *p* > 0.05; Supplementary Table S9). Therefore, laterality is presented as a secondary descriptive heterogeneity and prediction-modeling consideration rather than as a statistically confirmed treatment-effect modifier. Stroke-type-stratified models also showed numerically non-uniform coefficients (Figures 2D,E), but most confidence intervals overlapped substantially; statistically supported interaction evidence was limited to the timing association for FMA-LE gain (interaction beta = −0.459, *p* = 0.012; Supplementary Table S9). Anatomy-level analyses were retained as descriptive. Brainstem involvement showed nominally higher BI and FMA-UE gain in present-versus-absent contrasts, whereas anatomy-by-side home-discharge rates occupied a relatively narrow range across small strata (Figures 2F–H; Supplementary Figure S2). These patterns may help motivate heterogeneity-aware prediction, but they should not be read as proof that lesion side or anatomy changes the biological response to acupuncture.

### 3.5 Secondary endpoints, missingness, and missingness-aware sensitivity analyses

The secondary endpoints required more cautious interpretation because observation was incomplete (Supplementary Table S1; Supplementary Figure S8; Supplementary Figure S5). BI and the FMA domains were fully observed in all 1,048 patients, but MoCA was available in only 587 patients (43.99% missing) and ROM/Pain in 752 patients (28.24% missing; Supplementary Table S1; Supplementary Figure S8C; Supplementary Figure S5A). Even so, both outcomes improved within the observed subset: MoCA increased by 1.93 points and ROM/Pain by 1.60 points (Table 3; Supplementary Figures S8A,B). MoCA change decreased across later timing quartiles (trend *p* = 0.015), whereas no meaningful trend was seen across frequency quartiles (trend *p* = 0.911); ROM/Pain showed no significant timing gradient (trend *p* = 0.388; Supplementary Figures S8D–F). In model-based complete-case and observation-IPW analyses, ROM/Pain was also not associated with weekly acupuncture frequency (complete-case *p* = 0.726; IPW *p* = 0.630; Supplementary Table S3; Supplementary Figure S5F). Observation models clarified the missingness structure. In both MoCA and ROM/Pain, the strongest determinant of having paired observations was baseline motor status (Supplementary Table S2; Supplementary Figure S8I; Supplementary Figures S5B–D). For ROM/Pain, earlier timing and longer length of stay also increased the odds of being observed, whereas weekly acupuncture frequency and electroacupuncture status were not major drivers of observation (Supplementary Table S2; Supplementary Figures S5C,D). In complete-case models, earlier timing remained associated with greater MoCA improvement (beta = −0.209, *p* = 0.010), and this association strengthened after observation-IPW (beta = −0.276, *p* = 0.003). In contrast, ROM/Pain timing and frequency estimates were not significant in either complete-case or IPW analyses (Supplementary Table S3; Supplementary Figures S8G,H; Supplementary Figures S5E,F). These results support retaining MoCA as a cautious supportive exploratory domain, whereas ROM/Pain is best treated as exploratory because the observed improvement was not accompanied by a consistent timing or frequency association and the endpoint was incompletely captured.

### 3.6 Prediction, interpretability, and clinical scenario translation

**Figure 5:**
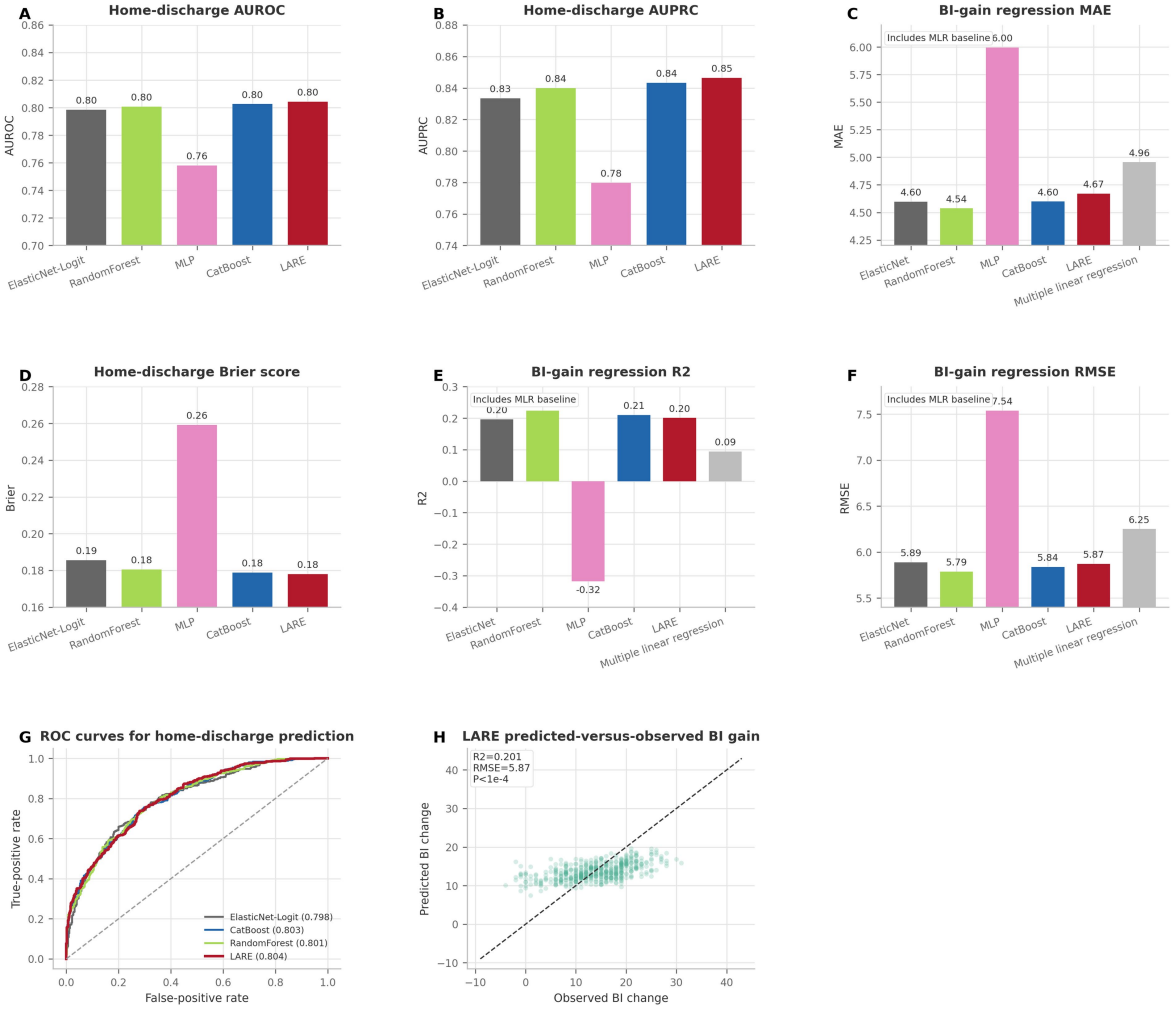
Benchmarking of predictive models for home discharge and BI improvement. **(A,B)** AUROC and AUPRC for 3-fold internally validated home-discharge prediction across ElasticNet-Logit, RandomForest, MLP, CatBoost, and LARE. LARE had the highest point estimates, but bootstrap intervals and pairwise tests in Table 4 show that it was not statistically distinguishable from CatBoost or RandomForest. **(C,E,F)** BI-gain regression MAE, R2, and RMSE comparisons across benchmarked algorithms, including a multiple-linear-regression baseline. RandomForest had the best point-estimate regression performance; bootstrap comparisons in Table 5 show that R2 and RMSE differences from CatBoost and LARE were not statistically significant, while MAE favored RandomForest over LARE. **(D)** Brier score comparison for home-discharge classification. **(G)** ROC curves for home-discharge prediction. **(H)** Predicted-versus-observed BI gain for LARE, showing moderate regression performance and weaker accuracy at the two outcome extremes. Overall, the figure supports stronger classification than regression performance and shows that leading classification models were similar within internal validation.

**Figure 6:**
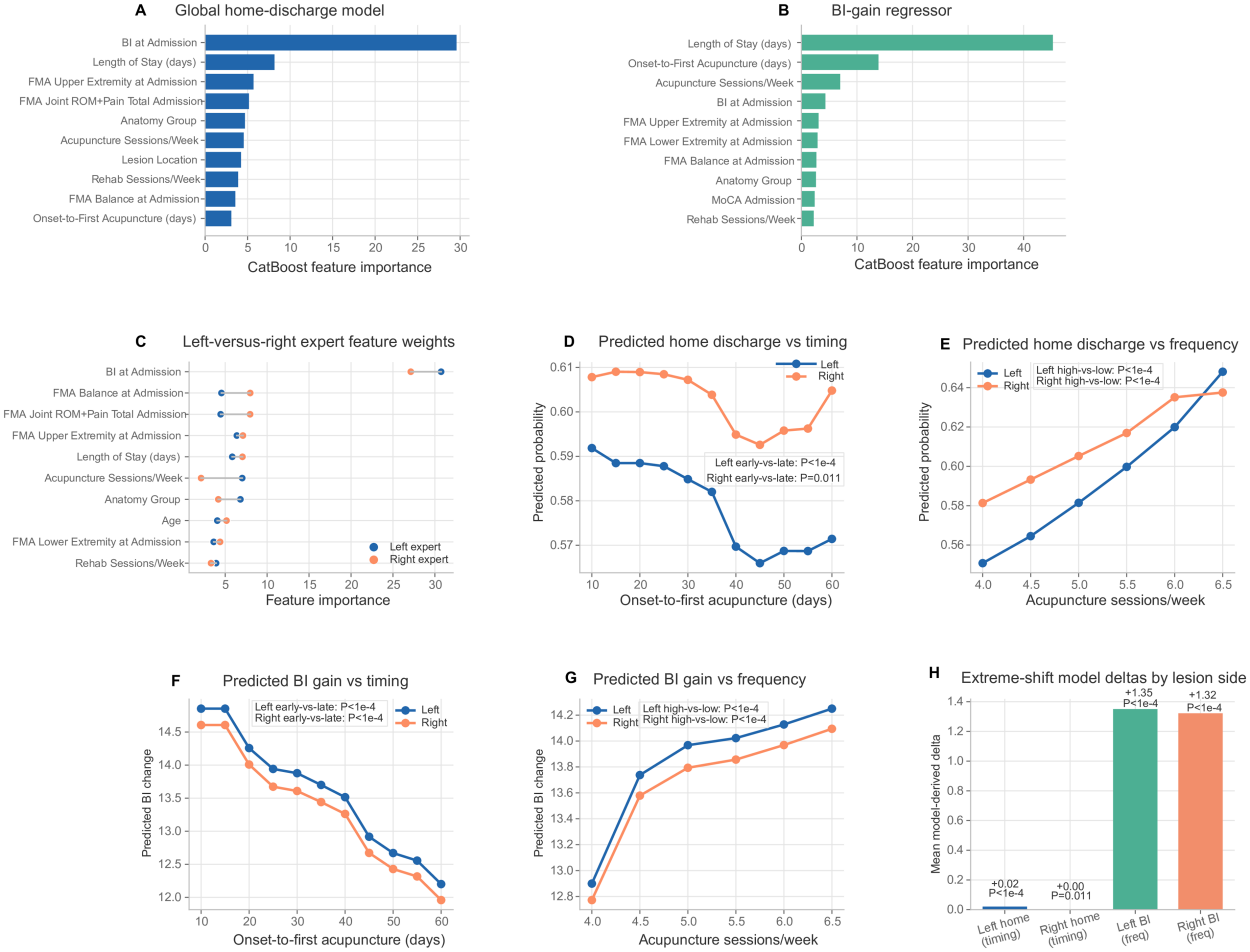
Exploratory interpretability of the laterality-stratified prediction benchmark. **(A)** Feature importance of the global home-discharge model. **(B)** Feature importance of the BI-gain regressor. **(C)** Left-versus-right expert feature weights from the lesion-side-specific expert models. **(D,E)** Predicted home-discharge probability across timing and weekly-frequency ranges by lesion side. **(F,G)** Predicted BI gain across timing and weekly-frequency ranges by lesion side. These curves were calculated by varying timing or weekly frequency across clinically observed ranges while holding other covariates at observed or reference values within lesion-side strata. **(H)** Mean model-derived deltas by lesion side, defined as the average predicted change between extreme early-versus-late timing and high-versus-low weekly-frequency settings. These panels are exploratory diagnostics for a benchmark model that did not show statistically supported superiority over standard tree-based models. Absolute left–right differences were small and are shown as model-derived prediction patterns, not as statistically confirmed group differences or causal treatment simulations.

Model benchmarking showed that internally validated classification performance for home discharge was consistently better than regression performance for BI gain (Tables 4, 5; Figure 5). Home discharge was selected because it is a practical rehabilitation endpoint that affects discharge planning, caregiver training, equipment preparation, post-acute resource allocation, and follow-up intensity; it is not a direct functional recovery score. For home-discharge classification, LARE had the highest point estimates (AUROC 0.804, 95% bootstrap CI 0.778–0.829; AUPRC 0.846, 95% CI 0.818–0.871; Brier 0.178, 95% CI 0.166–0.190), but these values were not statistically distinguishable from CatBoost (AUROC *p* = 0.348, AUPRC *p* = 0.177, Brier *p* = 0.257) or RandomForest (AUROC *p* = 0.321, AUPRC *p* = 0.163, Brier *p* = 0.170). Therefore, the laterality-aware residual blend did not provide a statistically supported improvement over standard tree-based benchmarks, although it performed better than the MLP model (Table 4; Figures 5A,B,D,G). For BI-gain regression, RandomForest had the strongest point-estimate performance (R2 0.224, 95% CI 0.168–0.276; RMSE 5.79, 95% CI 5.50–6.07; MAE 4.54, 95% CI 4.32–4.76). Differences in R2 and RMSE between RandomForest and CatBoost or LARE were not statistically significant, although RandomForest had lower MAE than LARE (*p* = 0.001; Table 5). A transparent multiple-linear-regression baseline using the main covariates reached only R2 = 0.094, RMSE = 6.25, and MAE = 4.96 (Table 5), supporting nonlinear benchmarking while also showing that BI-gain prediction remained limited. The observed-versus-predicted BI-gain plot showed weaker calibration at the two outcome extremes, so regression outputs should be interpreted as approximate risk stratification rather than precise individual recovery forecasts (Figure 5H; Supplementary Figures S3D,E). Calibration and decision-curve analyses supported the internal translational plausibility of the leading classification models but did not establish one model family as definitively superior. We retained the LARE interpretability panels as exploratory side-aware diagnostics, not as independent evidence of statistically significant left–right treatment differences or as proof that laterality-aware modeling is preferable (Figure 6). In the global home-discharge model, admission BI and length of stay carried the largest feature importance, followed by baseline FMA domains, ROM/Pain, anatomy group, lesion location, and weekly acupuncture frequency (Figure 6A). In the BI-gain regressor, length of stay and onset-to-first acupuncture timing were dominant features, with weekly acupuncture frequency, baseline BI/FMA, anatomy, and MoCA also contributing (Figure 6B). The lesion-side expert panel showed small model-derived differences in feature weighting (Figure 6C). Figures 6D–G show counterfactual predictions obtained by varying timing or weekly frequency across observed clinical ranges while holding other covariates at observed or reference values within lesion-side strata; Panel 6H reports mean model-derived deltas, defined as the average predicted change between extreme early-versus-late timing and high-versus-low frequency settings. The scenario curves have been moved to the Supplementary Figure S9 because they are illustrative decision-support visualizations rather than primary inferential results. The three displayed scenarios were selected to span clinically common contrasts in age, stroke type, lesion side, baseline severity, and comorbidity burden; they are not intended to exhaustively represent all combinations such as right-ischemic younger, left-hemorrhage, or younger multimorbid profiles.

**Table 4:** Home-discharge classification performance across benchmarked algorithms.

Model	AUROC (95% CI)	AUPRC (95% CI)	Brier (95% CI)	Bootstrap *p* values
ElasticNet-Logit	0.798 (0.771–0.823)	0.833 (0.802–0.862)	0.186 (0.172–0.200)	vs LARE: *p* = 0.414/*p* = 0.092/*p* = 0.051
RandomForest	0.801 (0.774–0.827)	0.840 (0.809–0.866)	0.180 (0.170–0.191)	vs LARE: *p* = 0.321/P = 0.163/P = 0.170
MLP	0.758 (0.728–0.787)	0.780 (0.740–0.818)	0.259 (0.236–0.283)	vs LARE: *p* < 1e-4/P < 1e-4/P < 1e-4
CatBoost	0.803 (0.776–0.827)	0.843 (0.814–0.869)	0.179 (0.167–0.192)	vs LARE: *p* = 0.348/P = 0.177/P = 0.257
LARE	0.804 (0.778–0.829)	0.846 (0.818–0.871)	0.178 (0.166–0.190)	Reference for pairwise bootstrap tests

Performance metrics are based on 3-fold internal validation. Larger AUROC and AUPRC are better; lower Brier score is better. Values in parentheses are 2,000-resample bootstrap 95% CIs from out-of-fold predictions. Pairwise *p* values are ordered as AUROC/AUPRC/Brier and compare each model against LARE.

**Table 5:** BI-gain regression performance across benchmarked algorithms.

Model	R2 (95% CI)	RMSE (95% CI)	MAE (95% CI)	Bootstrap *p* values
ElasticNet	0.196 (0.135–0.253)	5.888 (5.602–6.171)	4.597 (4.377–4.817)	vs RandomForest: *p* = 0.052/P = 0.052/*p* = 0.228
RandomForest	0.224 (0.168–0.276)	5.787 (5.499–6.071)	4.537 (4.318–4.756)	Reference for pairwise bootstrap tests
MLP	−0.318 (−0.446 to −0.205)	7.539 (7.203–7.861)	5.996 (5.722–6.260)	vs RandomForest: *p* < 1e-4/*p* < 1e-4/*p* < 1e-4
CatBoost	0.210 (0.171–0.247)	5.838 (5.555–6.116)	4.599 (4.378–4.821)	vs RandomForest: *p* = 0.269/P = 0.269/*p* = 0.155
LARE	0.201 (0.165–0.235)	5.872 (5.598–6.144)	4.669 (4.450–4.888)	vs RandomForest: *p* = 0.136/P = 0.136/P = 0.001
Multiple linear regression	0.094	6.250	4.955	Point-estimate reference

For BI-gain regression, larger R2 is better, whereas lower RMSE and MAE indicate lower prediction error. Values in parentheses are 2,000-resample bootstrap 95% CIs from out-of-fold predictions where available. Pairwise p values are ordered as R2/RMSE/MAE and compare each machine-learning model against RandomForest. The multiple linear-regression row is retained as a transparent point-estimate reference using the main covariates.

### 3.7 Focused robustness analysis of the right-lesion electroacupuncture signal

Because electroacupuncture showed a negative BI coefficient in the right-lesion subgroup (Table 6), we performed focused treatment-allocation imbalance and robustness analyses (Supplementary Tables S5, S6; Supplementary Figure S6). Treatment-component count was defined as the number of acupuncture-related modalities recorded for a patient; a higher count therefore indicates a more diverse and complex acupuncture-related treatment package rather than greater intensity of electroacupuncture itself. Among right-lesion patients, those receiving electroacupuncture differed from those not receiving electroacupuncture in treatment-component count (SMD 1.66; *p* < 0.001), warm-needle moxibustion exposure (SMD 0.39; *p* < 0.001), auricular acupuncture exposure (SMD 0.21; *p* = 0.025), admission MoCA (SMD 0.21; *p* = 0.098), onset-to-first acupuncture timing (*p* = 0.036), and several smaller variables such as hypertension (*p* = 0.205; Supplementary Tables S5; Supplementary Figures S6E–G). These treatment-context and case-mix differences argue against a simple causal interpretation of the electroacupuncture coefficient. Plausible explanations include indication-related allocation, in which clinicians may have selected electroacupuncture or broader multi-modality acupuncture packages for patients perceived as more complex, patients with slower early functional response, or patients requiring more symptom-directed treatment; such allocation could make electroacupuncture appear detrimental even if it was a marker of clinical complexity rather than the cause of lower BI gain. Nevertheless, the negative BI association was not a one-model artifact. In the right-lesion subset, the electroacupuncture coefficient for BI gain was negative in the unadjusted model (beta = −1.16, *p* = 0.066), base-adjusted model (beta = −1.58, *p* = 0.010), rehab-intensity-adjusted model (beta = −1.31, *p* = 0.020), baseline-domain-adjusted model (beta = −1.34, *p* = 0.018), and IPTW-based models (Supplementary Tables S6; Supplementary Figure S6C). However, the signal was outcome-specific: it did not generalize to FMA-UE gain, FMA-LE gain, or home discharge, and the crude difference in home-discharge rate by electroacupuncture status within right-sided lesions was not significant (55.7% vs. 57.8%, *p* = 0.722; Supplementary Tables S6; Supplementary Figures S6A–D). The most defensible interpretation is therefore that this is a right-lesion-specific, BI-focused, hypothesis-generating association potentially driven by treatment allocation bias or residual confounding rather than evidence of causal harm.

**Table 6:** Prespecified subgroup coefficients by lesion side and stroke type.

Grouping variable	Group	Outcome	Predictor	Coefficient	CI low	CI high	*p* value
Lesion Side	Left	BI gain	timing z	−1.708	−2.321	−1.095	<0.0001
Lesion Side	Left	BI gain	frequency z	1.308	0.767	1.849	<0.0001
Lesion Side	Left	BI gain	electro	−0.195	−1.426	1.037	0.757
Lesion Side	Left	FMA-UE gain	timing z	−0.650	−1.081	−0.219	0.003
Lesion Side	Left	FMA-UE gain	frequency z	0.683	0.197	1.168	0.006
Lesion Side	Left	FMA-UE gain	electro	0.042	−1.150	1.235	0.944
Lesion Side	Left	FMA-LE gain	timing z	−0.322	−0.554	−0.090	0.006
Lesion Side	Left	FMA-LE gain	frequency z	0.423	0.158	0.688	0.002
Lesion Side	Left	FMA-LE gain	electro	−0.242	−0.848	0.364	0.434
Lesion Side	Left	FMA-balance gain	timing z	−0.257	−0.469	−0.046	0.017
Lesion Side	Left	FMA-balance gain	frequency z	0.001	−0.198	0.199	0.994
Lesion Side	Left	FMA-balance gain	electro	0.027	−0.424	0.478	0.905
Lesion Side	Right	BI gain	timing z	−1.319	−1.966	−0.672	<0.0001
Lesion Side	Right	BI gain	frequency z	0.996	0.363	1.629	0.002
Lesion Side	Right	BI gain	electro	−1.480	−2.777	−0.183	0.025
Lesion Side	Right	FMA-UE gain	timing z	−0.514	−1.018	−0.010	0.046
Lesion Side	Right	FMA-UE gain	frequency z	0.410	−0.204	1.024	0.191
Lesion Side	Right	FMA-UE gain	electro	−0.336	−1.506	0.835	0.574
Lesion Side	Right	FMA-LE gain	timing z	−0.460	−0.747	−0.174	0.002
Lesion Side	Right	FMA-LE gain	frequency z	0.123	−0.189	0.435	0.440
Lesion Side	Right	FMA-LE gain	electro	−0.320	−0.944	0.304	0.315
Lesion Side	Right	FMA-balance gain	timing z	−0.305	−0.512	−0.098	0.004
Lesion Side	Right	FMA-balance gain	frequency z	0.020	−0.184	0.223	0.849
Lesion Side	Right	FMA-balance gain	electro	−0.427	−0.855	0.001	0.051
Stroke Type	Ischemic	BI gain	timing z	−1.369	−1.807	−0.932	<0.0001
Stroke Type	Ischemic	BI gain	frequency z	1.201	0.722	1.679	<0.0001
Stroke Type	Ischemic	BI gain	electro	−0.706	−1.728	0.315	0.175
Stroke Type	Ischemic	FMA-UE gain	timing z	−0.450	−0.791	−0.110	0.010
Stroke Type	Ischemic	FMA-UE gain	frequency z	0.758	0.326	1.189	0.0006
Stroke Type	Ischemic	FMA-UE gain	electro	−0.194	−1.114	0.725	0.679
Stroke Type	Ischemic	FMA-LE gain	timing z	−0.319	−0.503	−0.135	0.0007
Stroke Type	Ischemic	FMA-LE gain	frequency z	0.295	0.075	0.515	0.008
Stroke Type	Ischemic	FMA-LE gain	electro	−0.459	−0.940	0.022	0.061
Stroke Type	Ischemic	FMA-balance gain	timing z	−0.232	−0.387	−0.076	0.004
Stroke Type	Ischemic	FMA-balance gain	frequency z	0.104	−0.054	0.262	0.197
Stroke Type	Ischemic	FMA-balance gain	electro	−0.228	−0.575	0.119	0.198
Stroke Type	Hemorrhage	BI gain	timing z	−2.220	−3.596	−0.843	0.002
Stroke Type	Hemorrhage	BI gain	frequency z	1.418	0.750	2.086	<0.0001
Stroke Type	Hemorrhage	BI gain	electro	−1.182	−2.787	0.424	0.149
Stroke Type	Hemorrhage	FMA-UE gain	timing z	−0.738	−1.997	0.521	0.251
Stroke Type	Hemorrhage	FMA-UE gain	frequency z	0.391	−0.264	1.045	0.242
Stroke Type	Hemorrhage	FMA-UE gain	electro	−0.093	−1.689	1.503	0.909
Stroke Type	Hemorrhage	FMA-LE gain	timing z	−0.782	−1.133	−0.431	<0.0001
Stroke Type	Hemorrhage	FMA-LE gain	frequency z	0.472	0.112	0.833	0.010
Stroke Type	Hemorrhage	FMA-LE gain	electro	0.396	−0.459	1.250	0.364
Stroke Type	Hemorrhage	FMA-balance gain	timing z	−0.214	−0.552	0.123	0.213
Stroke Type	Hemorrhage	FMA-balance gain	frequency z	0.014	−0.243	0.272	0.913
Stroke Type	Hemorrhage	FMA-balance gain	electro	−0.179	−0.774	0.417	0.557

Subgroup coefficients are shown for standardized timing, weekly frequency, and electroacupuncture terms across BI, FMA-UE, FMA-LE, and balance outcomes. Formal coefficient-difference interaction tests are reported in Supplementary Table S9.

## 4 Discussion

This study supports a three-part clinical story. First, acupuncture-treated stroke rehabilitation inpatients showed marked within-stay improvement, with the clearest signal in ADL and motor outcomes rather than in secondary cognitive or ROM/Pain domains. Because every patient received acupuncture-related care and no non-acupuncture comparator was available, this within-stay improvement cannot be separated from the broader rehabilitation program. Second, within this acupuncture-treated setting, earlier initiation, weekly frequency, and total treatment exposure were associated with recovery patterns, especially for BI and the motor scales. Third, these associations were heterogeneous across clinical strata in descriptive analyses, but formal interaction tests showed that only selected subgroup coefficient differences were statistically supported.

The principal contribution of this dataset is therefore not a comparative estimate of acupuncture efficacy. Rather, its value lies in showing that treatment-pattern variation inside an acupuncture-integrated inpatient rehabilitation pathway is measurable, clinically coherent, and potentially useful for optimizing practice and discharge planning.

The timing signal was the most robust result across the manuscript. Earlier initiation was associated with larger gains in BI, FMA-UE, FMA-LE, balance, and composite recovery in the whole cohort, and the direction of effect remained stable across lesion-side and stroke-type subgroup analyses as well as extended sensitivity models. Frequency-related associations were also important, but they were more selective: they were strongest for BI improvement and home discharge and less consistent for balance and some subgroup-specific motor outcomes. These findings align with the broader rehabilitation literature emphasizing dose and timing (4–7) and with recent acupuncture-after-stroke evidence suggesting that treatment window, course length, and cumulative sessions may influence limb-function outcomes (13). Mechanistically, basic and translational acupuncture studies have proposed effects on neuroplasticity, cerebral perfusion, inflammatory signaling, and motor-network remodeling after ischemic injury (36). These literatures support biological plausibility for timing and dose hypotheses, but they do not eliminate the residual confounding inherent in the present retrospective cohort.

The heterogeneity results should be interpreted cautiously. The left–right lesion analyses suggested different coefficient patterns, especially for weekly frequency, but formal interaction testing did not confirm statistically significant left-versus-right coefficient differences. Stroke-type interaction testing provided stronger evidence only for the timing association with FMA-LE gain. Anatomical variables have also been reported to influence acupuncture response in earlier CT lesion-site work (37), and our nominal brainstem/cerebellum findings are compatible with the clinical importance of corticospinal, postural-control, and arousal networks. However, the present anatomy-level results were descriptive and based on small strata, so they should be used to generate hypotheses rather than to claim anatomy-specific efficacy. Importantly, these data do not establish lesion side as a treatment-effect modifier, and the laterality-aware ensemble did not show statistically supported superiority over standard CatBoost or RandomForest benchmarks. LARE should therefore be read as a neutral exploratory test of whether side-specific expert blending adds incremental prediction, not as a central positive methodologic finding. The dominant role of baseline functional status in our home-discharge model is also consistent with classic stroke-rehabilitation discharge-prediction studies, where early functional measures repeatedly emerged as the strongest determinants of return home or institutional care (38, 39).

The secondary outcomes deserve a more cautious reading. MoCA and ROM/Pain improved in the observed subsets, but the missingness burden was too high for them to carry equal inferential weight with BI and FMA. The observation models showed that missingness was related mainly to baseline severity and hospital-stay structure, which is clinically plausible, and the timing association for MoCA persisted after observation-IPW. Even so, these results should remain supportive and exploratory, not headline findings.

The right-lesion electroacupuncture result also requires cautious interpretation. The association with lower BI gain persisted across multiple model specifications, but it was entangled with treatment-package diversity and case-mix differences and did not generalize to FMA-UE, FMA-LE, FMA-balance, or home discharge. Accordingly, it should be presented as a hypothesis-generating signal that merits future prospective evaluation, not as evidence that electroacupuncture is harmful in right-sided lesions.

The prediction results are clinically useful but should not be overstated. Home-discharge classification around AUROC 0.80 with reasonable calibration is credible and potentially useful for adjunct discharge planning, family counseling, and resource preparation, while BI-gain regression around R2 0.20 is moderate and weaker at the two observed outcome extremes. The bootstrap analyses show that small point-estimate differences among the leading classification models should not be interpreted as proof of one algorithm’s superiority. Model choice for future implementation should therefore prioritize external validation, calibration, clinical workflow fit, and interpretability rather than marginal internal ranking differences.

The study has several strengths: a relatively large inpatient cohort, multiple functional endpoints, a coherent full-cohort-to-subgroup-to-prediction analysis chain, explicit attention to missingness, calibration, robustness, total-session sensitivity, and formal subgroup interaction tests. It also has important limitations. First, there was no non-acupuncture comparator, so the study cannot estimate the causal efficacy of acupuncture. Second, the retrospective single-center design leaves potential residual confounding and treatment-allocation bias. Third, NIHSS at stroke onset or acupuncture initiation was not available; baseline BI and FMA scores captured functional severity at rehabilitation admission but not acute neurologic deficit. Fourth, total acupuncture sessions were available and analyzed, but they were strongly correlated with length of stay, so their association may partly reflect exposure opportunity rather than treatment dose alone. Fifth, admission-to-discharge change scores can be affected by length of stay, baseline severity, and recovery opportunity; sensitivity analyses addressed these issues only partially. Sixth, discharge destination is clinically important but heterogeneous: transfer to another hospital or a nursing home may reflect premorbid support, institutional policy, caregiver availability, or prolonged care needs rather than recovery alone. Seventh, MoCA and ROM/Pain were incompletely captured. Eighth, no external validation cohort was available for the prediction models.

## 5 Conclusion

In acupuncture-treated stroke rehabilitation inpatients, earlier initiation, weekly treatment frequency, and total acupuncture exposure were associated with better short-term ADL and motor recovery, but these associations were observational and should be interpreted with caution. Formal interaction testing supported only selected subgroup differences, and laterality-aware prediction did not show a statistically supported advantage over standard tree-based benchmarks. The manuscript is therefore best positioned as an observational study of treatment-pattern optimization, sensitivity analysis, secondary heterogeneity, and clinical prediction rather than a causal comparative-efficacy analysis or a positive laterality-specific modeling claim.

## Data Availability

The raw data supporting the conclusions of this article will be made available by the authors, without undue reservation.
